# SARS-CoV-2 Achieves Immune Escape by Destroying Mitochondrial Quality: Comprehensive Analysis of the Cellular Landscapes of Lung and Blood Specimens From Patients With COVID-19

**DOI:** 10.3389/fimmu.2022.946731

**Published:** 2022-07-01

**Authors:** Chenyang Duan, Ruiyan Ma, Xue Zeng, Bing Chen, Dongyao Hou, Ruixue Liu, Xuehan Li, Liangming Liu, Tao Li, He Huang

**Affiliations:** ^1^ Department of Anesthesiology, The Second Affiliated Hospital of Chongqing Medical University, Chongqing, China; ^2^ Department of Cardiovascular Surgery, Xinqiao Hospital, Army Medical University, Chongqing, China; ^3^ Department of Shock and Transfusion, State Key Laboratory of Trauma, Burns and Combined Injury, Daping Hospital, Army Medical University, Chongqing, China

**Keywords:** COVID-19, mitochondrial quality, immune escape, inflammation, cytokine storm

## Abstract

Mitochondria get caught in the crossfire of coronavirus disease 2019 (COVID-19) and antiviral immunity. The mitochondria-mediated antiviral immunity represents the host’s first line of defense against viral infection, and the mitochondria are important targets of COVID-19. However, the specific manifestations of mitochondrial damage in patients with COVID-19 have not been systematically clarified. This study comprehensively analyzed one single-cell RNA-sequencing dataset of lung tissue and two bulk RNA-sequencing datasets of blood from COVID-19 patients. We found significant changes in mitochondrion-related gene expression, mitochondrial functions, and related metabolic pathways in patients with COVID-19. SARS-CoV-2 first infected the host alveolar epithelial cells, which may have induced excessive mitochondrial fission, inhibited mitochondrial degradation, and destroyed the mitochondrial calcium uniporter (MCU). The type II alveolar epithelial cell count decreased and the transformation from type II to type I alveolar epithelial cells was blocked, which exacerbated viral immune escape and replication in COVID-19 patients. Subsequently, alveolar macrophages phagocytized the infected alveolar epithelial cells, which decreased mitochondrial respiratory capacity and activated the ROS–HIF1A pathway in macrophages, thereby aggravating the pro-inflammatory reaction in the lungs. Infected macrophages released large amounts of interferon into the blood, activating mitochondrial IFI27 expression and destroying energy metabolism in immune cells. The plasma differentiation of B cells and lung-blood interaction of regulatory T cells (Tregs) was exacerbated, resulting in a cytokine storm and excessive inflammation. Thus, our findings systematically explain immune escape and excessive inflammation seen during COVID-19 from the perspective of mitochondrial quality imbalance.

## Introduction

Coronavirus disease 2019 (COVID-19) is an acute respiratory syndrome caused by severe acute respiratory syndrome coronavirus 2 (SARS-CoV-2) infection (1) Patients with COVID-19 manifest a range of varying severity, ranging from no symptoms (asymptomatic) to severe pneumonia, which can progress to acute respiratory distress syndrome, metabolic acidosis, septic shock, coagulopathy, organ failure, and even death (2). According to the World Health Organization, the cumulative numbers of confirmed cases and deaths reported worldwide have exceeded 433 and 5.9 million until April 2022, respectively, since its outbreak in 2019 (3, 4). The disease is still spreading with more than 5,00,000 new confirmed cases and nearly 20,000 deaths per day. Thus, the spread of SARS-CoV-2 is still a serious Public Health Emergency of International Concern. Therefore, it is essential to investigate the pathological reactions involved in and immune mechanisms underlying the effects of COVID-19.

SARS-CoV-2 is an enveloped positive-strand single-stranded RNA virus of the family Coronaviridae, which binds to human angiotensin-converting enzyme 2 or human dipeptidyl peptidase 4 with its receptor-binding domain of the S protein (5). After SARS-CoV-2 enters the cell, it efficiently replicates and produces offspring. In patients with COVID-19, the most common presentation in the chest computed tomography scan is ground glass shadows distributed in one or both peripheral lung and subpleural regions, and reticular and/or interlobular septal thickening and consolidation (6). These signs are closely related to the alveolar edema and inflammatory response caused by SARS-CoV-2 infection (7). Tissue biopsies and autopsies have revealed pathological manifestations such as alveolar edema, hyaline membrane formation, multinucleated enlarged cell deposition, and diffuse thickening of the alveolar wall in the lungs of COVID-19 patients (8).

Recent studies have suggested that COVID-19 should be considered a vascular disease (9). In addition to lung injury, patients with COVID-19 also show obvious characteristics of vascular damage, including endothelial cell inflammation in the pulmonary artery, extensive thrombosis, and microvascular lesions (2). Electron microscopic analysis has shown the presence of virus particles in lymphocytes and vascular endothelial cells, indicating that the vascular injury and blood immune response may be directly related to cytotoxic invasion by the virus (10). Cytokine storm (CS) has also been observed in the blood of patients with moderate or severe COVID-19; strong systemic symptoms have been noted in such cases. In the absence of timely and effective treatment, CS can lead to the systemic inflammatory syndrome, multiple organ dysfunction, and even death (11). Many cytokines, such as interleukin (IL)-6, IL-1, IL-10, tumor necrosis factor-α, and interferon (IFN)-γ (12), and various types of cells, including macrophages, neutrophils, eosinophils, lymphocytes, and basophils, are involved in CS (13). However, the relationship between the CS in the blood and the lung injury caused by SARS-CoV-2 has not yet been clarified.

Mitochondria are organelles that constitute key components of human innate immunity and energy metabolism. Mitochondrial quality control factors, including mitochondrion-related gene expression, mitochondrial functions, and related metabolic pathways, regulate the processing and turnover of native proteins to control protein import, signaling cascades, mitochondrial dynamics, lipid biogenesis the proper function of mitochondria. Thus, mitochondrial quality control mechanisms are important in integrating mitochondria into the cellular environment (14). Recent research has shown that SARS-CoV-2 can inhibit the innate immune response of the human body and that mitochondria comprise one of the first lines of defense against SARS-CoV-2 infection (15). Compared with other viruses, such as respiratory syncytial virus, seasonal influenza A virus, and human parainfluenza virus, SARS-CoV-2 is the only virus that can reduce mitochondrion-related protein expression (16). SARS-CoV-2 can colonize mitochondria and interact with mitochondrial protein translocation mechanisms to target its coding products to the mitochondria (17); this process enables inhibition of the degradation of viral proteins and host misfolded proteins (including mitochondrial proteins) and is, therefore, crucial for viral replication and escape from host innate immunity (16). This phenomenon partly explains why COVID-19 causes more severe effects in older people and people with mitochondrial metabolic dysfunction (17). Although studies (17, 18) have reported that mitochondria are intricately involved in COVID-19 etiopathogenesis, the specific manifestations of mitochondrial damage in patients with this disease and the relationship between this damage and COVID-19 occurrence and development have not been systematically clarified.

This study aimed to clarify the process involved in immunopathological changes, such as immune escape, and the cell fate in COVID-19 by studying the expression and translocation of mitochondrion-related genes in lung tissue and the process involved in the lung–blood interaction in COVID-19. Therefore, we performed in-depth analysis of a set of single-cell datasets of the lung tissue as well as two sets of transcriptome datasets of the peripheral blood from COVID-19 patients and systematically detected the changes in mitochondrion-related gene expression, mitochondrial functions as well as related metabolic pathways to lay the foundation on using mitochondrial quality control as the main intervention for COVID-19.

## Materials and Methods

### Processing of Data Obtained From the Gene Expression Omnibus Dataset

Single-cell RNA-seq datasets pertaining to human lung tissue were obtained from a Gene Expression Omnibus (GEO) dataset (GSE171524), including data for frozen lung specimens obtained from 19 patients with COVID-19 and 7 control patients for whom short postmortem interval autopsies had been performed. Two RNA-seq datasets pertaining to human blood samples were obtained from two GEO datasets (GSE157103 and GSE152641). The GSE157103 dataset includes data for 126 plasma and leukocyte samples from hospitalized patients with or without COVID-19 (n = 100 and 26, respectively). The GSE152641 dataset includes data for peripheral blood samples from 24 healthy controls and 62 prospectively enrolled patients with community-acquired lower respiratory tract infection by SARS-CoV-2 within the first 24 h of hospital admission.

### Single-Cell RNA-Seq Data Processing

Single-cell RNA-seq data were analyzed using a NovaSeq 6000 sequencing system (Illumina, USA). Unique molecular identifier tools were used to identify the whitelist of the cell barcodes. Single-cell gene expression matrices were generated using the Celescope software (version 1.7.2, Singleron, Germany). The transcripts were aligned to the human GRCh38 reference genome, which was appended with the entire SARS-CoV-2 genome (GenBank, MN908947.3) as an additional chromosome to the human reference genome (19). Cells with more than 200 detected genes and a mitochondrial unique molecular identifier rate of less than 30% were considered to have passed cell quality control. Subsequently, principal component analysis was performed for the scaled data to determine the top 2,000 highly variable genes and top 10 principal components, as well as for tSNE and UMAP construction (20). The main cell types were identified by manual annotation of the genes differentially expressed between the clusters. The positive markers for each cluster were identified on the basis of a previous study (19), with a minimal fraction of 25% and log-transformed fold change threshold of 0.25.

### Bulk RNA-Seq Data Processing

Raw reads were trimmed using the GeneChem cloud analysis platform. The trimmed reads were mapped to the hg19 genome using HISAT2 (version 2.0.4), thereby generating sam files, which were then converted to bam files using SAMtools (version 1.6). HTSeq (version 0.11.0) was used to calculate the read count for each gene. DEGs were identified using the R package DESeq2 (version 1.26.0), with a cutoff of adjusted p-value < 0.05 and |log2FC| > 1.

### Annotation of Gene Functions

To investigate the potential biological functions of mitochondrial DEGs in individuals with or without COVID-19, the “clusterProfiler” package (version 3.16.1) in R was used to perform the GO analysis, KEGG analysis, and GSEA (21). We performed GSVA using the “GSVA” R package to estimate the biological function of different clusters, which estimates the variations in pathway activity over a sample population in an unsupervised manner (22). Significantly enriched pathways were filtered using an adjusted p-value of <0.05.

### Estimation of Immune Cell Fractions

The abundance of immune cells was determined by cell type identification *via* “CIBERSORT,” an algorithm that combines support vector regression from purified leukocyte subsets. The bulk RNA-seq data were uploaded to the CIBERSORT website (https://cibersortx.stanford.edu/), and the LM22 signature gene matrix served as an input of the “CIBERSORT” algorithm. Correlations between gene expression and the immune infiltrate abundance were estimated using the “Gene” module. Additionally, the “SCNA” module was used to examine the correlation between somatic copy number alterations and immune infiltrate abundance.

### Statistical Analysis

The statistical analysis was performed using R (version 3.6.1; R Foundation for Statistical Computing, Vienna, Austria), complemented by IBM SPSS Statistics 24.0 (IBM, Inc., Armonk, NY, USA). All statistical tests were two-sided, and a p-value <0.05 was considered statistically significant. Other statistical methods were described within the related results and Figure legends.

## Results

### The Cellular Landscape of Lung Tissue From Patients With COVID-19

**Figure 1:**
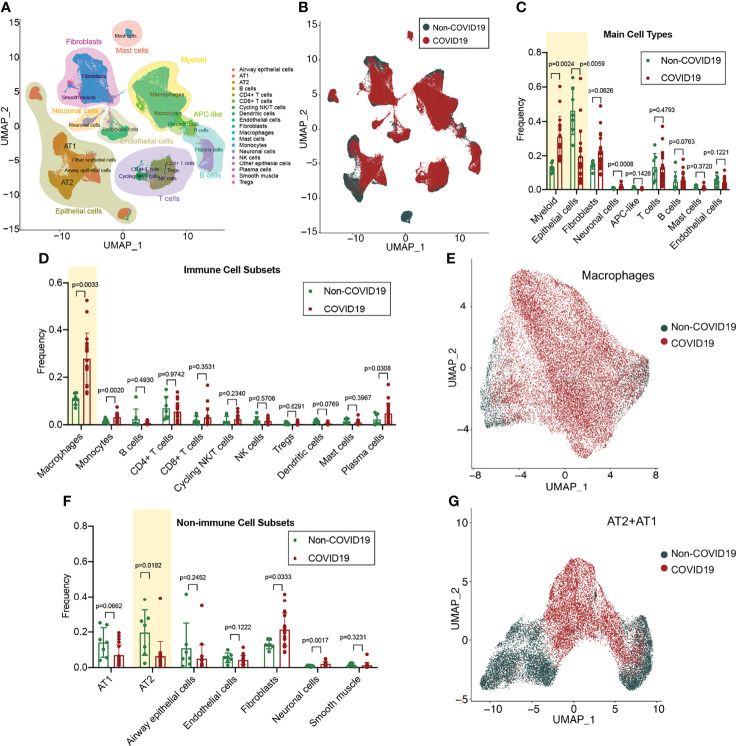
Single-cell atlas and cell subset fractions in the lung tissue from COVID-19 patients. **(A)** Main clusters and respective cell-type assignments in the uniform manifold approximation and projection (UMAP). **(B)** Origins of cells with same embedding in COVID-19 (n = 19) and non-COVID-19 (n = 7) lungs. **(C)** Fraction of main cell types in COVID-19 and non-COVID-19 lungs. **(D)** Fraction of immune cell subsets in COVID-19 and non-COVID-19 lungs. **(E)** UMAP and corresponding group assignments of the macrophages analyzed. **(F)** Fraction of non-immune cell subsets in COVID-19 and non-COVID-19 lungs. **(G)** UMAP and corresponding group assignments of the epithelial cells analyzed, including AT2 and AT1.

The cell types in the lung tissue are complex. For in-depth analysis of lung tissue lesions from patients with COVID-19, we selected a single-cell RNA-sequencing (RNA-seq) dataset of lung tissue from patients with COVID-19 (GSE171524), which includes data on the frozen lung specimens obtained from 19 patients with COVID-19 and 7 control patients with short postmortem interval autopsies. We identified nine main cell types by using the uniform manifold approximation and projection (UMAP) method: epithelial cells (n = 30,070 cells), myeloid cells (n = 29,632 cells), fibroblasts (n = 22,909 cells), endothelial cells (n = 5,386 cells), T and natural killer lymphocytes (n = 16,751 cells), lymphocytes and plasma cells (n = 7,236 cells), neuronal cells (n = 2,017 cells), mast cells (n = 1,464 cells), and antigen-presenting cells (primarily dendritic cells; n = 849 cells) (**Figure 1A**). The disease sorting results showed that the cell types and counts in the lung tissue of COVID-19 patients significantly differed from those of non-COVID-19 patients (**Figure 1B**). In the lung tissue of patients with severe COVID-19, the myeloid cell count significantly increased (p=0.0024) and the epithelial cell count significantly decreased (p=0.0059) (**Figure 1C**). Further analysis of immune-related cell subsets revealed that the increase in myeloid cells was mainly due to the large increase in the alveolar macrophage count (p=0.0033) (**Figure 1D**) and that the macrophage functions in the COVID-19 group may also be significantly different from those in the non-COVID-19 group (**Figure 1E**). Assays of non-immune–related cell subsets indicated that the decrease in epithelial cells was mainly due to the considerable decrease in type II alveolar epithelial cell (AT2) count (p=0.0182) (**Figure 1F**); it was found that the transformation from AT2 to type I alveolar epithelial cells (AT1) may be blocked in the lung tissue of patients with COVID-19 (**Figures 1F** **, G**).

### Aberrant Macrophage Transformation Was Influenced by Mitochondrial Dysfunction in the Lung Tissue of COVID-19 Patients

**Figure 2:**
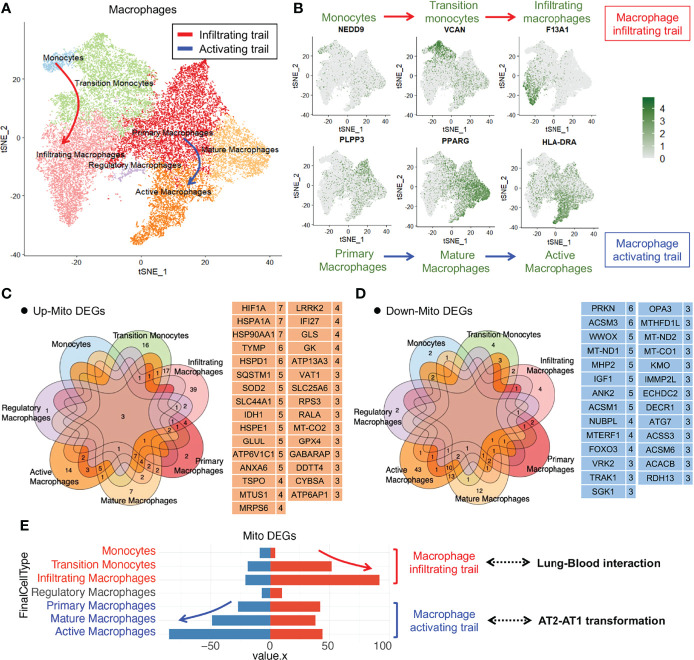
Immune macrophage transformation in the lung tissue from COVID-19 patients. **(A)**T-distributed stochastic neighbor embedding (tSNE) projection highlighting immune macrophage transformation trails. Red arrow represents macrophage infiltrating trail and blue arrow represents macrophage activating trail. **(B)** tSNE of representative markers RNA expression showing macrophage transformation trails. **(C)** Upregulated mitochondrial differentially expressed genes (mito-DEGs) of macrophage subsets in COVID-19. **(D)** Downregulated mito-DEGs of macrophage subsets in COVID-19. **(E)** Statistical analysis of mito-DEGs, accompanied by the transformation of macrophage infiltrating and activating trails.

To analyze the changes in the alveolar macrophage count and functions in COVID-19 lung tissues, we performed dimensionality reduction clustering and subgroup annotation of the macrophages. Seven subsets were identified using the t-distributed stochastic neighbor embedding (tSNE) method: monocytes, transition monocytes, infiltrating macrophages, regulatory macrophages, primary macrophages, mature macrophages, and active macrophages (**Figure 2A**). The first three subsets belonged to the macrophage infiltrating trail, whereas the last three subsets belonged to the macrophage activating trail (**Figure 2B**).

To analyze the mitochondrial damage in the alveolar macrophages of patients with COVID-19, we first searched for all mitochondrion-encoding genes and mitochondrial function–regulating genes from the Gene Set Enrichment Analysis (GSEA), Gene Cards, and UniProt databases and merged them into a complete mitochondrion-related gene cluster comprising 1,513 genes (**Table S1**). We screened mitochondrial differentially expressed genes (mito-DEGs) in at least 3 macrophage subsets and identified 31 upregulated mito-DEGs (HIF1A, HSPA1A, HSP90AA1, TYMP, HSPD1, SQSTM1, SOD2, SLC44A1, IDH1, HSPE1, GLUL, ATP6V1C1, ANXA6, TSPO, MTUS1, MRPS6, LRRK2, IFI27, GLS, GK, ATP13A3, VAT1, SLC25A6, RPS3, RALA, MT-CO2, GPX4, GABARAP, DDTT4, CYBSA, and ATP6AP1) (**Figure 2C**) and 27 downregulated mito-DEGs (PRKN, ACSM3, WWOX, MT-ND1, MHP2, IGF1, ANK2, ACSM1, NUBPL, MTERF1, FOXO3, VRK2, TRAK1, SGK1, OPA3, MTHFD1L, MT-ND2, MT-CO1, KMO, IMMP2L, ECHDC2, DECR1, ATG7, ACSS3, ACSM6, ACACB, and RDH13) (**Figure 2D**). Gene expression analysis of macrophage subsets revealed that the mito-DEGs in the macrophage infiltrating trail were gradually upregulated, whereas those in the macrophage activating trail were gradually downregulated (**Figure 2E**). These results suggested that alveolar macrophage transformation is closely related to the expression of mitochondrion-related genes in patients with COVID-19.

**Figure 3:**
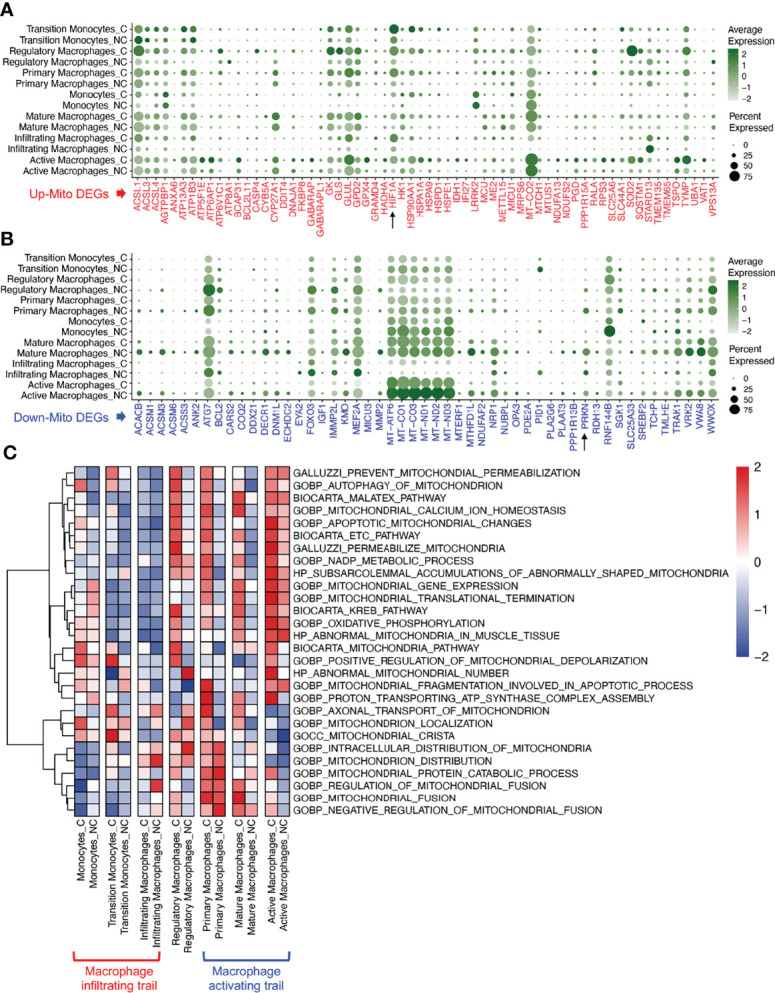
Mitochondrial functions of macrophage subsets in COVID-19 lung tissues. **(A)** Bubble chart of upregulated mito-DEGs of macrophage subsets in COVID-19. **(B)** Bubble chart of downregulated mito-DEGs of macrophage subsets in COVID-19. **(C)** GSVA of mitochondrion-related pathways in macrophage subsets in COVID-19.

The changes in mito-DEGs in different macrophage subsets are directly reflected in the bubble chart provided in **Figure 3A**; after SARS-CoV-2 infection, *HIF1A* expression significantly increased and *PRKN* expression significantly decreased (**Figure 3B**) in all the macrophage subsets. Based on these mito-DEGs, we further analyzed the mitochondrial functions of macrophage subsets by using GSEA-based pathway enrichment analysis. The key pathways involved in mitochondrial quality regulation, such as mitochondrial oxidative phosphorylation, mitochondrial autophagy, and apoptotic mitochondrial changes, were generally enriched in most macrophage subsets in patients with COVID-19 (**Figure S1**). Gene set variation analysis (GSVA) further showed that the mitochondrial pathway activity in the macrophage activating trail was much higher in patients with COVID-19 (**Figure 3C**). Activated macrophages are a subset of innate alveolar macrophages; they have antigen-presenting characteristics and can phagocytize virus-infected alveolar epithelial cells, which may play an important role in the repair of lung tissue injury. The mitochondrial pathway activity in the macrophage infiltrating trail was lower in patients with COVID-19 (**Figure 3C**). Infiltrating macrophages may belong to the exogenous macrophage subsets produced by the lung’s damaged blood immune cells.

### Impaired Alveolar Epithelial Regeneration Was Influenced by Excessive Mitochondrial Fission and MCU Destruction in the Lung Tissue of COVID-19 Patients

**Figure 4:**
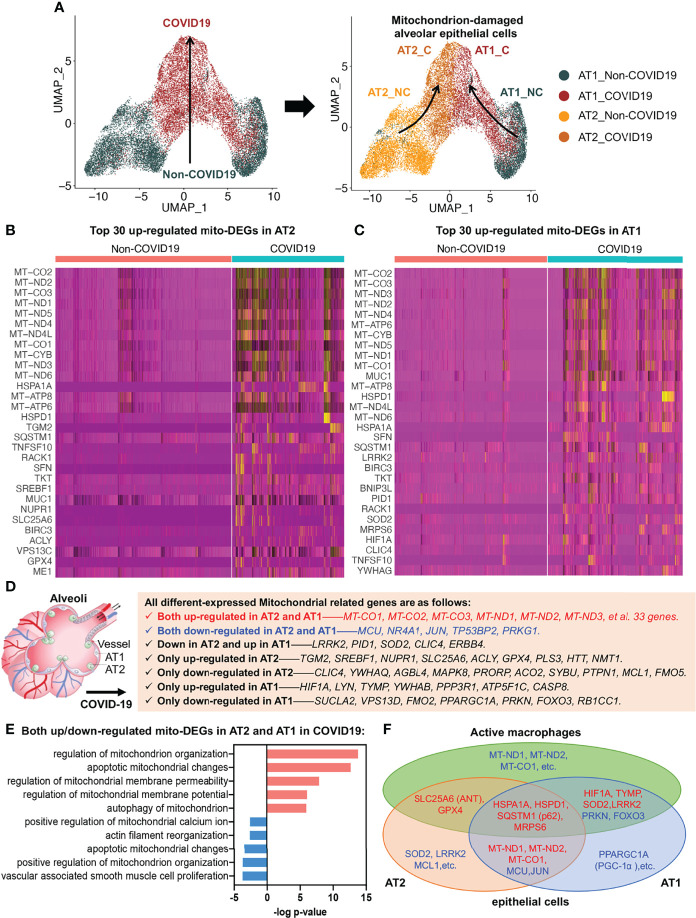
Alveolar epithelial regeneration in COVID-19 lung tissues. **(A)** UMAP and corresponding group assignments of AT2 and AT1 in COVID-19. C: COVID-19 group; NC: Non-COVID-19 group. **(B)** Heat map of top 30 upregulated mito-DEGs in AT2 during COVID-19. **(C)** Heat map of top 30 upregulated mito-DEGs in AT1 during COVID-19. **(D)** Statistical analysis of mito-DEGs in AT2 and AT1 during COVID-19. **(E)** GO pathway enrichment analysis in relation to mito-DEGs in AT2 and AT1 during COVID-19. **(F)** Venn diagram showing the intersection mito-DEGs from AT2, AT1, and active macrophages during COVID-19.

To investigate alveolar epithelial regeneration in patients with COVID-19, we performed subset analysis and found that the two typical alveolar epithelial cell subsets, AT2 and AT1, were clearly distinguished in the non-COVID-19 group; by contrast, in patients with COVID-19, the cellular properties of the AT2 and AT1 subsets gradually converged, leading to their transformation to mitochondrion-damaged alveolar epithelial cells (**Figure 4A**).

To examine the link between alveolar epithelial regeneration and mitochondrial damage in patients with COVID-19, we separately analyzed the mito-DEGs of the AT2 and AT1 subsets in patients with COVID-19 (**Figures 4B** **, C**). We identified 38 mito-DEGs that showed consistent changes in expression in the AT2 and AT1 subsets in patients with COVID-19. The mito-DEGs upregulated in AT2 and AT1 included the following: 13 mitochondrion-coding genes (mitochondrial COX subunits, i.e., MT-CO1, MT-CO2, and MT-CO3; mitochondrial NADH subunits, i.e., MT-ND1, MT-ND2, MT-ND3, MT-ND4, MT-ND4L, MT-ND5, and MT-ND6; and ATP synthases, i.e., MT-ATP6, MT-ATP8, and MT-CYB) and 20 mitochondrial function regulatory genes (HSPD1, HSPA1A, SQSTM1, MUC1, SFN, BIRC3, TKT, BNIP3L, RACK1, MRPS6, TNFSF10, YWHAG, CTTN, NDUFS1, MRPS25, ME1, XIAP, MRPL14, BCL2L1, and VPS13C). The mito-DEGs showing downregulation in the AT2 and AT1 subsets included MCU, TP53BP2, JUN, PRKG1, and NR4A1. In addition, some mito-DEGs were reversely altered in the AT2 and AT1 subsets (e.g., LRRK2, PID1, SOD2, CLIC4, and ERBB4) or showed altered expression in the AT2 or AT1 subset in patients with COVID-19 (**Figures 4B** **–D**). It should be noted that the general upregulation of mitochondrion-encoding genes suggested that the alveolar epithelial cells of patients with COVID-19 would have excessive mitochondrial fission, resulting in a large increase in the number of damaged mitochondria, and the decrease in MCU expression suggested that the mitochondrial calcium transport channels of alveolar epithelial cells were disrupted in patients with COVID-19 (**Figure 4D**).

Functional enrichment analysis also established that several key aspects of mitochondrial quality regulation, such as mitochondrial organization, apoptotic mitochondrial changes, mitochondrial membrane permeability, mitochondrial membrane potential, mitophagy, mitochondrial calcium homeostasis, and cytoskeleton regulation, were impaired to various degrees (**Figure 4E**). The Kyoto Encyclopedia of Genes and Genomes (KEGG) annotation results further showed that the damaged mitochondrial clearance and degradation pathways in alveolar epithelial cells were significantly inhibited, resulting in the accumulation of large amounts of mitochondrion-damaged alveolar epithelial cells in patients with COVID-19 (**Figure S2**). The accumulation of damaged mitochondria in the AT2 subset was mainly due to inhibition of apoptotic mitochondrial changes (**Figure S2A**), whereas the accumulation in the AT1 subset was mainly due to inhibition of the mitophagy pathway (**Figure S2B**).

The mito-DEGs in the AT2, AT1, and active macrophage subsets are shown in the Venn diagram in **Figure 4F**; the main genetic signatures of active macrophages that phagocytized virus-infected epithelial cells were found to be increased expression of HIF1A, TYMP, SOD2, LRRK2, HSPA1A, HSPD1, SQSTM1 (p62), MRPS6, SLC25A6 (ANT), GPX4 and decreased expression of PRKN, FOXO3. In addition, the decreased expression of mitochondrion-encoding genes, such as MT-ND1, MT-ND2, and MT-CO1, were also found in active macrophages after phagocytosis of virus-infected alveolar epithelial cells (**Figure 4F**).

### Irregular Distribution of Immune Cells Was Affected by the Mitochondrial IFI27-Mediated IFN Immune Response in the Blood Samples of COVID-19 Patients

**Figure 5:**
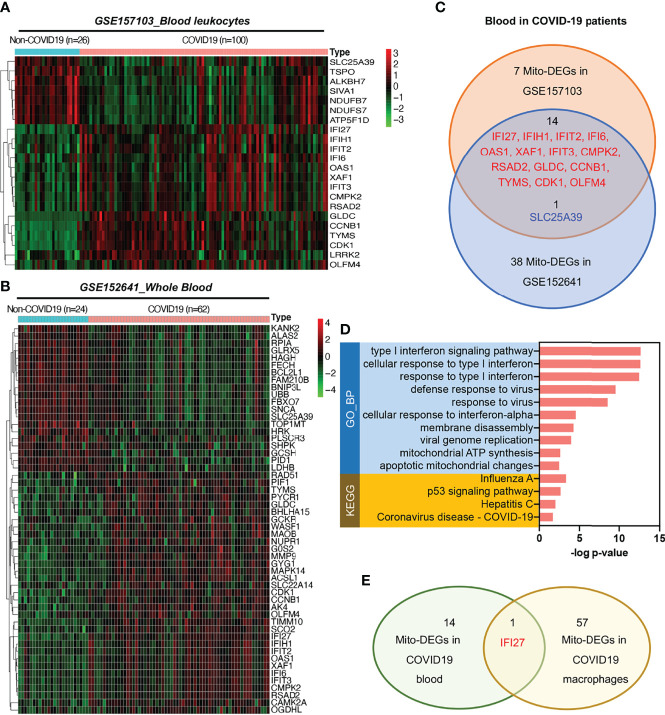
Expression of mitochondrion-related genes in the blood of patients with COVID-19. **(A)** Heat map of mito-DEGs in blood leukocytes from GSE157103. **(B)** Heat map of mito-DEGs in the whole blood from GSE152641. **(C)** Venn diagram showing the intersection mito-DEGs between these two GSE datasets. **(D)** Functional enrichment analysis of mito-DEGs in blood from patients with COVID-19. GO analysis and KEGG annotation are both listed. **(E)** Venn diagram showing the intersection mito-DEGs between blood and macrophages in COVID-19.

To analyze mitochondrial damage in the blood immune cells of patients with COVID-19, we selected two bulk RNA-seq datasets (GSE157103 and GSE152641) of peripheral blood from patients with this disease. The GSE157103 dataset contains information on the blood leukocyte samples from 100 patients with severe COVID-19 and 26 patients without COVID-19. The GSE152641 dataset contains information on the peripheral blood samples from 62 patients with COVID-19 and whole blood samples from 24 healthy controls. The differential expression analysis of mitochondrion-related genes is shown in **Figures 5A** **, B**. The Venn diagram shows that the following 14 mitochondrion-related genes were upregulated in the blood of patients with COVID-19 in both datasets: IFI27, IFIH1, IFIT2, IFI6, OAS1, XAF1, IFIT3, CMPK2, RSAD2, GLDC, CCNB1, TYMS, CDK1, and OLFM4 (**Figure 5C**). KEGG annotation showed that these significantly upregulated mitochondrion-related genes were closely related to the blood immune response after viral infection. Gene Ontology (GO) functional enrichment analysis showed that these significantly upregulated mitochondrion-related genes were closely related to viral immune response pathways, such as the type I IFN signaling pathway, the pathways involved in the response to virus and viral genome replication, and mitochondrial dysfunction pathways such as those involved in mitochondrial ATP synthesis and apoptotic mitochondrial changes (**Figure 5D**). Among the 14 mitochondrion-related genes showing upregulation in the blood samples, IFI27 showed significantly upregulated expression in the infiltrating macrophage subsets of patients with COVID-19 (**Figures 2C**, **5E**), suggesting that IFI27 is involved in the blood immune response and in the lung–blood interaction process after viral infection.

**Figure 6:**
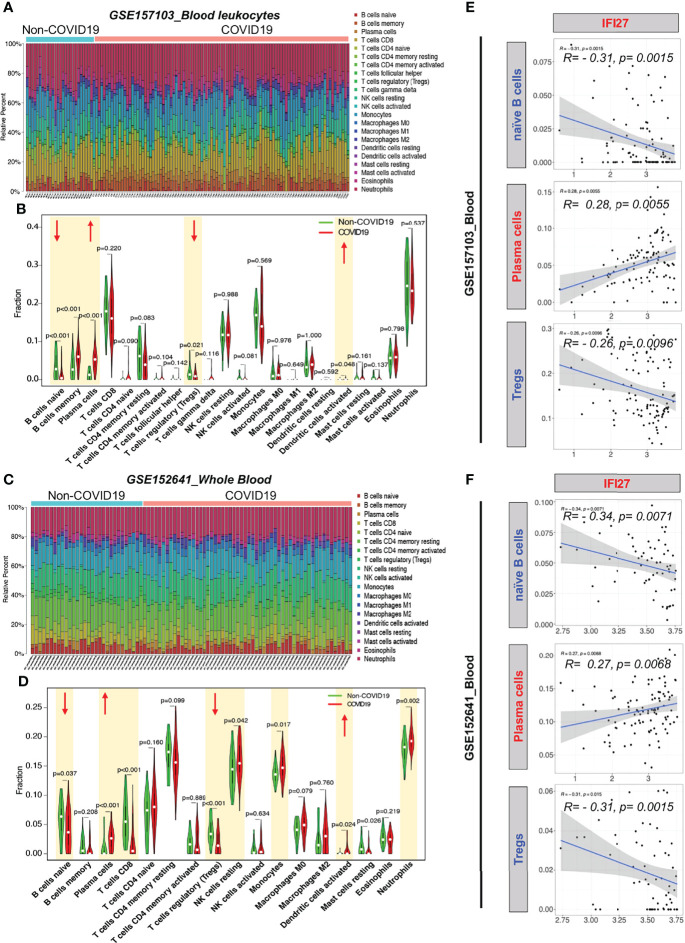
CIBERSORT analysis of immune cell count in the blood of patients with COVID-19. **(A)** Distribution proportion of immune cells in each sample from GSE157103. **(B)** Statistical analysis of various immune cell types in blood leukocytes from patients with COVID-19 and non-COVID-19 patients in GSE157103. **(C)** Distribution proportion of immune cells in each sample from GSE152641. **(D)** Statistical analysis of various immune cell types in whole blood from patients with COVID-19 and non-COVID-19 patients in GSE152641. **(E)** Correlation analysis between IFI expression and immune cell content in GSE157103. **(F)** Correlation analysis between IFI expression and immune cell content in GSE152641.

To examine the blood immune response of patients with COVID-19 and its relationship with IFI27, we used CIBERSORT to analyze the landscape of immune cells in the blood of patients with COVID-19. GSE157103 dataset analysis showed that the proportions of naïve B cells and regulatory T cells (Tregs) decreased, whereas the proportions of memory B cells, plasma cells, and activated dendritic cells increased in the blood of patients with COVID-19 (**Figures 6A** **, B**). Furthermore, GSE157103 dataset analysis showed that the proportions of naïve B cells, CD8^+^ T cells, and Tregs decreased, whereas those of plasma cells, resting natural killer cells, monocytes, activated dendritic cells, and neutrophils increased in the blood of patients with COVID-19 (**Figures 6C** **, D**).

To determine whether the IFI27-mediated IFN immune response affects the counts of blood immune cells in patients with COVID-19, we analyzed the correlation between IFI27 expression and the abovementioned significantly altered immune cell counts. IFI27 expression was negatively correlated with the naïve B cell and Treg counts and positively correlated with the plasma cell counts in the blood of patients with COVID-19 (**Figures 6E** **, F**). The abovementioned results suggested that IFI27 expression upregulation in the blood immune cells of patients with COVID-19 accelerated the differentiation of naïve B cells into plasma cells and the infiltration of Tregs into alveoli.

## Discussion

**Figure 7:**
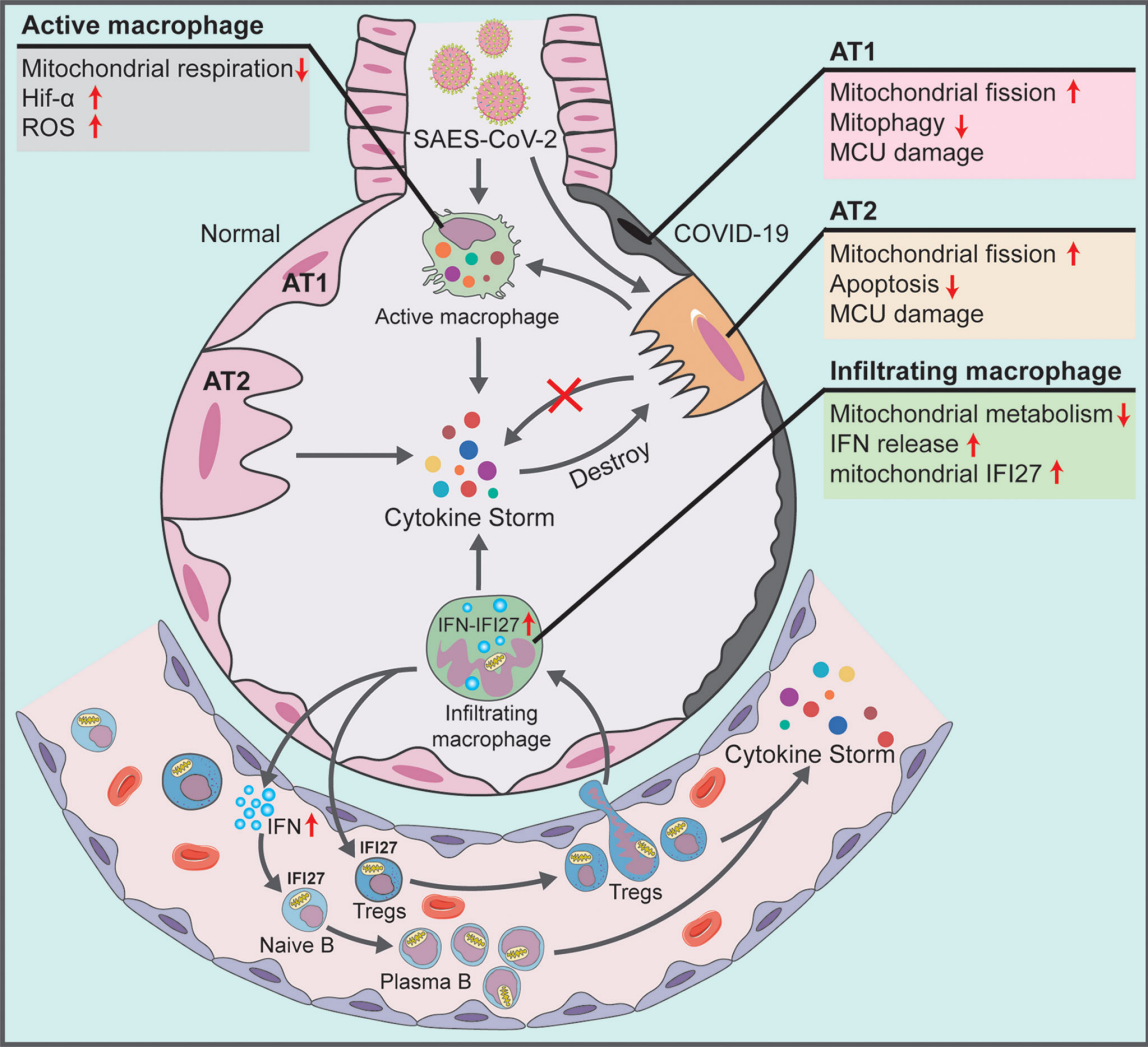
Schematic showing the systemic pathological changes in the lung and blood specimens of patients with COVID-19.

This study noted the severe mitochondrial quality imbalance in the lung tissue and blood immune cells of patients with COVID-19, which played a key role in viral immune escape and cell turnover. Our findings indicated that the mechanism underlying the damage caused by the virus was as follows: (1) In alveolar epithelial cells infected by SARS-CoV-2, there exists excessive mitochondrial fission, inhibition of mitochondrial degradation pathway and destruction of MCU calcium channel, thereby resulting in decreased AT2 counts and blocking the transformation of AT2 to AT1. The accumulation of many mitochondrion-damaged alveolar epithelial cells exhibit an impaired immune response, enhancing viral immune escape and replication. (2) In alveolar macrophages that swallow virus-infected alveolar epithelial cells or are directly infected by SARS-CoV-2, the low mitochondrial respiratory ability and the subsequently activated mitochondrial ROS-HIF1A pathway aggravate the pro-inflammatory response of lung tissue in patients with COVID-19. The infected alveolar macrophages further release high levels of IFN into the blood. (3) In blood immune cells of patients with COVID-19, the IFN release activates mitochondrial IFI27 and destroys mitochondrial energy metabolism in T and B cells, thereby promoting the plasma cell differentiation and lung–blood shuttle of Tregs, and eventually leading to cytokine storm and excessive inflammation (**Figure 7**).

Under normal circumstances, host cells rapidly respond to invading viruses by coordinating the functions of various organelles after viral infection (23). However, during highly invasive/aggressive viral infection, including that with SARS-CoV-2, the immune response function of organelles is controlled by the virus and is used to avoid the antiviral response, enhancing the ability of virus replication, and cause immune escape. Recent studies have shown that the viral structural proteins of SARS-CoV-2 can be translated and inserted into endoplasmic reticulum–mitochondrial contacts. Viral RNA synthesis is associated with endoplasmic reticulum and mitochondrial membrane modifications, and double-membrane vesicles produced by the endoplasmic reticulum and mitochondria may be central hubs for viral RNA synthesis and replication (24). Travaglini et al. detected the protein products of SARS-CoV-2 using affinity purified mass spectrometry and found that three nonstructural proteins (NSP4, NSP8, and ORF9c) were enriched in mitochondrial ribosomes, suggesting that mitochondria are one of the important target organelles of SARS-CoV-2, which disrupts host cell function (25). Miller et al. showed that SARS-CoV-2 could mask the reactive oxygen species (ROS) produced during cellular energy metabolism by inhibiting mitochondrial respiratory chain complex I, resulting in inability to activate cells to produce immune resistance (16). Ajaz et al. showed that abnormal mitochondrial pentose phosphate metabolism in host cells could enhance viral RNA replication and viral pathogenicity and help evade antiviral responses (26). In the current study, we found that multiple key factors affecting mitochondrial quality, such as mitochondrial coding and regulation of gene expression, mitochondrial function, and related metabolic pathways, were impaired to different degrees in various types of tissue cells in patients with COVID-19, indicating that mitochondria play a critical role in the immune response of host cells to SARS-CoV-2.

We found significant changes in the expression of mitochondrion-related genes in the lung tissue and blood of patients with COVID-19. The specific alterations were as follows: In alveolar macrophages, the expression of 31 mitochondrion-related genes such as HIF1A, SQSTM1, SOD2, IDH1, TSPO, LRRK2, and GPX4 was upregulated and the expression of 27 mitochondrion-related genes such as PRKN, ATG7, FOXO3, MT-ND1, MT-ND-2, MT-CO1, and OPA3 was downregulated. In lung epithelial cells, the genes that were simultaneously upregulated in the AT2 and AT1 subtypes included 13 mitochondrion-encoding genes (e.g., genes encoding the COX subunit, NADH subunit, and ATP synthase, etc.) and 20 mitochondrial function–regulating genes (e.g., HSPD1, HSPA1A, SQSTM1 and BNIP3L, etc.); the genes that were simultaneously downregulated in the AT2 and AT1 subtypes included MCU, TP53BP2, JUN, PRKG1, and NR4A1. In blood leukocytes, 14 mitochondrion-related genes, namely, IFFI27, IFIH1, IFIT2, IFI6, OAS1, XAF1, IFIT3, CMPK2, RSAD2, GLDC, CCNB1, TYMS, CDK1, and OLFM4, were upregulated. Our study systematically describes the specific changes that occurred in mitochondrion-related genes in the lung tissue of patients with COVID-19 and during lung–blood interaction. It also provides data supporting the requirement for further analysis of the important role of and specific regulatory mechanism underlying mitochondrial quality imbalance in immune escape and cell outcome in patients with COVID-19.

SARS-CoV-2 has been reported to first invade ciliated cells in the proximal airways and AT2 cells in the gas-exchange zone of the distal lung (5, 27). AT2 cells are progenitor cells of the lung epithelium and precursor cells of AT1 and can, therefore, regulate lung epithelial cell homeostasis *via* self-renewal and differentiation, maintain alveolar tension by secreting pulmonary surfactant, and support efficient gas exchange at the lung–blood interface (28). After mild inflammatory stimulation or viral infection, AT2 cells can present antigens to T cells to initiate T cell responses (29) and maintain alveolar function by increasing citrate synthase expression, upregulating the expression of the mitochondrial biogenesis–related gene PPARGC1A (PGC-1α), and promoting mitochondrial apoptosis and other approaches for maintaining alveolar function (30). However, our current study showed that the AT2 count significantly decreased in the lung tissue of patients with COVID-19. The conversion of AT2 to AT1 was blocked, which may be an important reason for the body’s failure to mount an immune response against SARS-CoV-2. In addition, mitochondrial calcium uptake plays an important role in the conversion of AT2 to AT1, and MICU1-MCU channels represent important mechanisms for regulating the differentiation of AT2 to AT1 cells (31). In our current study, we found that MCU expression was significantly downregulated in both AT2 and AT1 cells in patients with COVID-19, suggesting that the destruction/blocking of MCU channels in alveolar epithelial cells by SARS-CoV-2 infection may be a potential mechanism underlying the blocking of AT2 to AT1 conversion.

Our study showed that the expression of mitochondrion-encoding genes was upregulated and that of the mitochondrial biogenesis–related gene PPARGC1A (PGC-1α) was downregulated in the alveolar epithelial cells of patients with COVID-19. These findings suggested that the mitochondria in alveolar epithelial cells from such patients exhibit excessive fission without additional biogenesis, resulting in a considerable increase in the number of damaged mitochondria. Moreover, our findings showed that, after SARS-CoV-2 infection, multiple key aspects of mitochondrial quality regulation, such as mitochondrial morphological features, mitochondrial apoptosis, and mitochondrial membrane potential, are affected in lung epithelial cells. In patients with COVID-19, the JUN-MCL1-mediated mitochondrial apoptosis pathway is inhibited in AT2 cells and the PRKN-FOXO3-mediated mitophagy pathway is inhibited in AT1 cells. The abovementioned results suggested that, because damaged mitochondria cannot be effectively degraded and cleared *via* apoptosis or mitophagy, many mitochondrion-damaged lung epithelial cells cannot initiate the damage repair mechanism of the host in the lung epithelial cells of patients with COVID-19, resulting in COVID-19 immune escape, which accelerates the viral replication process.

Alveolar macrophages are yolk sac–derived heterogeneous mononuclear phagocytes with complex ontogeny and reside in the alveolus pulmonis. When infection is initiated, these cells are responsible for early pathogen identification, inflammation initiation and regression, and tissue damage and repair (32). In alveolar tissue, cleaning of residual, such as virus or damaged cells, depends on macrophages to swallow and transfer to local lymph nodes, thus triggering immune protection (33). However, in the lung tissue of patients with severe COVID-19, macrophages have been found to be infected by SARS-CoV-2, as indicated by the presence of viral nucleocapsid proteins (34), and these macrophages aggravate the damage in concert with the virus itself (35). In our current study, we found that HIF1A expression significantly increased in the alveolar macrophages of patients with COVID-19. Zhu et al. (36) also noted upregulation of HIF1A expression in the alveolar lavage fluid of patients with COVID-19, consistent with our findings. High HIF1A levels can promote glycolysis in alveolar macrophages, inhibit mitochondrial function, and aggravate proinflammatory responses and lung tissue damage (37). In virally infected animal and cell models, inhibiting the HIF1A pathway can promote the proliferation of reparative macrophages, which is conducive to tissue repair and homeostasis after infection and increase in survival rate.

Our current study also showed that the alveolar macrophages in patients with COVID-19 have two developmental trajectories. The expression level of mitochondrion-related genes was upregulated, consistent with the maturation process of infiltrating macrophages, whereas that of active macrophages was gradually downregulated. Thus, SARS-CoV-2 infection may have a greater impact on the mitochondrial pathway activity of active macrophages. This effect is probably due to the recognition and phagocytosis of virus-infected lung epithelial cells; expression of mitochondrion-encoding genes such as MT-ND1, MT-ND2, and MT-CO1 decreased in active macrophages, whereas that of mitochondrion-regulatory genes such as SOD2, SQSTM1 (p62), and LRRK2 increased. The decreased expression of the mitochondrion-encoding genes suggested that mitochondrial spare respiratory capacity decreased in the alveolar macrophages of patients with COVID-19 (37). Furthermore, increased expression of the mitochondrion-regulatory genes, such as SOD2, SQSTM1 (p62), and LRRK2, activated the oxidative stress pathway of macrophages and led to production of large amounts of ROS, which helped stabilize HIF1A (38–40). These results suggest that activation of the macrophage ROS-HIF1A pathway due to phagocytosis of apoptotic lung epithelial cells or directly due to viral infection may be pivotal in aggravating the pro-inflammatory response in lung tissue.

Some studies have shown that macrophages infected with SARS-CoV-2 release T cell chemokines, thereby attracting many T cells into the lungs and promoting T cell activation and proliferation (35, 41). T cells produce large amounts of IFN, which induces the release of many inflammatory factors by alveolar macrophages into the blood. However, they also induce the death of infected macrophages and spread to the microvasculature to recruit monocytes into the lungs, where the cells rapidly differentiate into alveolar macrophages, thereby leading to positive feedback regulation. In our current study, we noted decrease in the counts of naïve B cells and Tregs and increase in the counts of plasma cells and activated dendritic cells in the blood of patients with COVID-19. The differential expression of mitochondrion-related genes in these cells suggests that this process may be closely related to the type I IFN immune response to virus. A large amount of IFN is produced by infected blood immune cells; it activates effectors such as IFI27 to fight the virus (42). In addition, IFI27, which is located in the mitochondria, plays an important role in regulating mitochondrial energy metabolism, such as the tricarboxylic acid (TCA) cycle and respiratory chain uncoupling (43). Our study showed that IFI27 expression significantly increased in alveolar invasive macrophage subsets and blood immune cells in patients with COVID-19 and played an important role in the disproportion of peripheral blood immune cells and lung–blood interaction. These results suggest that IFI27 can be used as a mitochondrial immune marker to evaluate the degree of viral infection and cell turnover in patients with COVID-19 (44).

Mitochondrial quality is extremely important for B cell transformation. When mitochondrial quality is excellent, B cells tend to undergo type transformation; however, when the mitochondrial quality is imbalance, they undergo differentiation to plasma cells (45). We found that the proportion of naïve B cells decreased and that of plasma cells increased in patients with COVID-19, suggesting that the mitochondrial damage to blood B cells caused by SARS-CoV-2 infection accelerates the differentiation of B cells to plasma cells (effector B cells). T cell dysregulation is closely related to COVID-19 severity. Tregs are regulatory CD4^+^ T cell subsets that maintain peripheral immune tolerance (46). Tregs play a key role in maintaining immune homeostasis and inhibiting excessive inflammatory responses by inducing other immune cell activation, proliferation, and effector functions (47). They have been found to alleviate virus-induced pneumonia and acute lung injury by inhibiting CS in respiratory virus infection (48, 49). On the basis of our findings, it can be presumed that the CS and excessive inflammatory response caused by the decrease in Tregs in the blood of patients with severe COVID-19 may be the main reasons for the poor prognosis of patients with severe COVID-19.

The limitations of this study are as follows: (1) Owing to the stringent experimental requirements of SARS-CoV-2–related research, we could not perform the relevant animal or cell experiments. (2) The current available public datasets have less accurate prognostic information and have scattered information regarding related biochemical indicators on patients with COVID-19, which are insufficient for determining the correlation between mitochondrial–related genes and organ functions or prognosis of patients with COVID-19. We plan to perform further research after identifying more detailed datasets with multiple organ indexes when available. (3) Currently, a SARS-CoV-2 mutant strain, that is, Omicron, has been identified (50). Further studies are required to determine whether the mutant viruses cause differing degrees of mitochondrial damage in the lung tissue and blood immune cells. We will continue to follow up on detailed information and related sequencing data of patients infected with the original virus or with different mutants.

In conclusion, our study emphasizes that mitochondria get caught in the crossfire of COVID-19 and immunity, and systematically explains the reasons for the immune escape and excessive inflammation noted during COVID-19 from the perspective of mitochondrial quality imbalance in lung tissue and lung–blood interaction. Further studies should be performed to develop mitochondrial quality control as the main intervention for functional protection of critically affected organs, for example, in the lungs of patients with COVID-19.

## Data Availability Statement

The datasets presented in this study can be found in online repositories. The names of the repository/repositories and accession number(s) can be found in the article/**Supplementary Material**.

## Ethics Statement

Ethical review and approval was not required for the study on human participants in accordance with the local legislation and institutional requirements. Written informed consent for participation was not required for this study in accordance with the national legislation and the institutional requirements.

## Author Contributions

CD designed the whole project. XZ and CD performed the single-cell RNA-seq data processing in this study. BC and RN performed the bulk RNA-seq data processing in this study. DH, RL and XL were responsible for literature search. CD wrote the manuscript and LL, TL, HH were responsible for manuscript editing and revision. CD and HH provided scientific research funding support. All authors read and approved the final manuscript.

## Funding

This work was supported by the China Postdoctoral Science Foundation (2021MD703924), Chongqing Postdoctoral Innovative Talents Support Program (CQBX2021018) and Kuanren Talents Program of the second affiliated hospital of Chongqing Medical University.

## Conflict of Interest

The authors declare that the research was conducted in the absence of any commercial or financial relationships that could be construed as a potential conflict of interest.

## Publisher’s Note

All claims expressed in this article are solely those of the authors and do not necessarily represent those of their affiliated organizations, or those of the publisher, the editors and the reviewers. Any product that may be evaluated in this article, or claim that may be made by its manufacturer, is not guaranteed or endorsed by the publisher.

## Supplementary Material

Supplementary Figure 1 GSEA-based pathway enrichment analysis in relation to the macrophage subsets in COVID-19. **(A)** Macrophage infiltrating trails, including monocytes, transition monocytes, and infiltrating macrophages. **(B)** Macrophage activating trails, including primary macrophages, mature macrophages, and active macrophages.

Supplementary Figure 2 Representative KEGG annotation in relation to mito-DEGs in AT2 and AT1 during COVID-19. **(A)** Apoptosis pathway (KEGG: hsa04210) enriched by mito-DEGs in AT2. **(B)** Mitophagy pathway (KEGG: hsa04137) enriched by mito-DEGs in AT1.

Supplementary Table 1 Cluster lists of 1,513 mitochondrion-related genes.
