# Supplementary figures and images for: SARS-CoV-2 Achieves Immune Escape by Destroying Mitochondrial Quality: Comprehensive Analysis of the Cellular Landscapes of Lung and Blood Specimens From Patients With COVID-19
Source: Front Immunol. 2022 Jul 1;13:946731. doi: 10.3389/fimmu.2022.946731 (PMC9283956; doi:10.3389/fimmu.2022.946731)

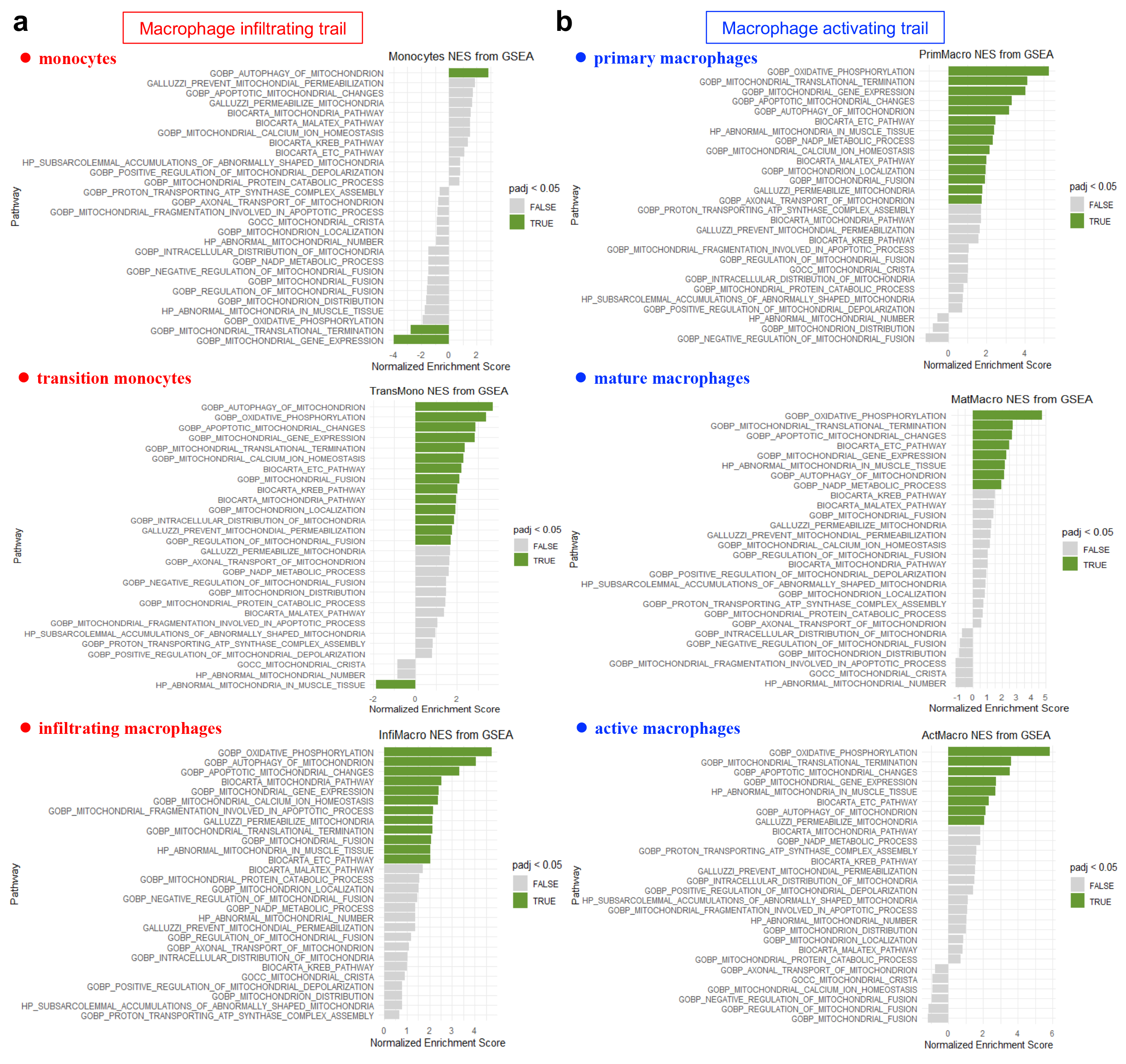

Supplement: Supplementary Figure 1 — GSEA-based pathway enrichment analysis in relation to the macrophage subsets in COVID-19. (A) Macrophage infiltrating trails, including monocytes, transition monocytes, and infiltrating macrophages. (B) Macrophage activating trails, including primary macrophages, mature macrophages, and active macrophages. [file Image_1.tif]

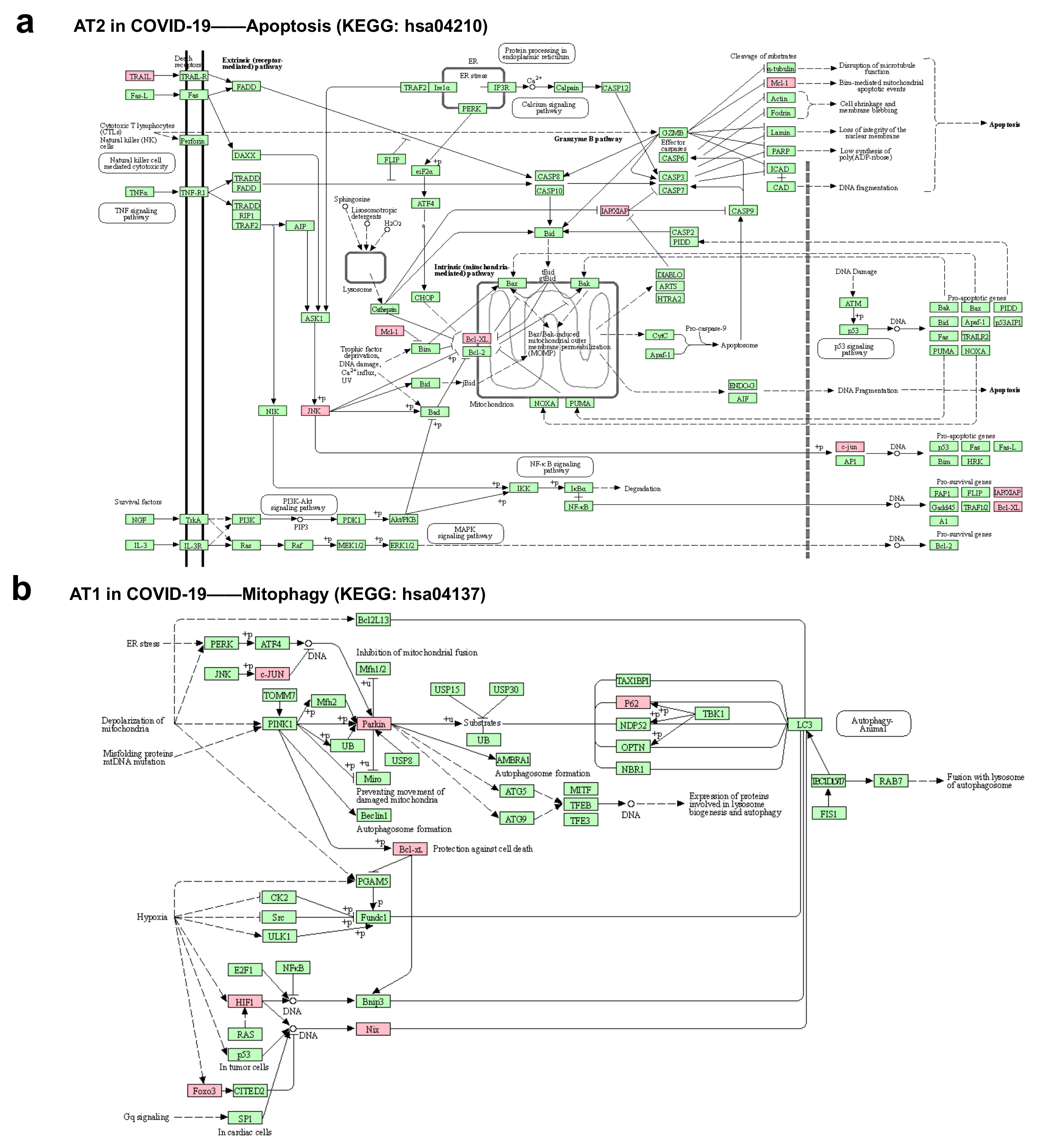

Supplement: Supplementary Figure 2 — Representative KEGG annotation in relation to mito-DEGs in AT2 and AT1 during COVID-19. (A) Apoptosis pathway (KEGG: hsa04210) enriched by mito-DEGs in AT2. (B) Mitophagy pathway (KEGG: hsa04137) enriched by mito-DEGs in AT1. [file Image_2.tif]
